# Design and Development of a Context-Aware Knowledge-Based Module for Identifying Relevant Information and Information Gaps in Patients With Type 1 Diabetes Self-Collected Health Data

**DOI:** 10.2196/10431

**Published:** 2018-07-11

**Authors:** Alain Giordanengo, Pinar Øzturk, Anne Helen Hansen, Eirik Årsand, Astrid Grøttland, Gunnar Hartvigsen

**Affiliations:** 1 Department of Computer Science The University of Tromsø - The Arctic University of Norway Tromsø Norway; 2 Norwegian Centre for E-health Research University Hospital of North Norway Tromsø Norway; 3 Department of Computer Science The Norwegian University of Science and Technology Trondheim Norway; 4 Centre for Quality Improvement and Development University Hospital of North Norway Tromsø Norway; 5 Department of Community Medicine The University of Tromsø - The Arctic University of Norway Tromsø Norway; 6 Department of Clinical Medicine The University of Tromsø - The Arctic University of Norway Tromsø Norway

**Keywords:** context aware, knowledge-based system, diabetes, self-collected health data, information gaps

## Abstract

**Background:**

Patients with diabetes use an increasing number of self-management tools in their daily life. However, health institutions rarely use the data generated by these services mainly due to (1) the lack of data reliability, and (2) medical workers spending too much time extracting relevant information from the vast amount of data produced. This work is part of the FullFlow project, which focuses on self-collected health data sharing directly between patients’ tools and EHRs.

**Objective:**

The main objective is to design and implement a prototype for extracting relevant information and documenting information gaps from self-collected health data by patients with type 1 diabetes using a context-aware approach. The module should permit (1) clinicians to assess the reliability of the data and to identify issues to discuss with their patients, and (2) patients to understand the implication their lifestyle has on their disease.

**Methods:**

The identification of context and the design of the system relied on (1) 2 workshops in which the main author participated, 1 patient with type 1 diabetes, and 1 clinician, and (2) a co-design session involving 5 patients with type 1 diabetes and 4 clinicians including 2 endocrinologists and 2 diabetes nurses. The software implementation followed a hybrid agile and waterfall approach. The testing relied on load, and black and white box methods.

**Results:**

We created a context-aware knowledge-based module able to (1) detect potential errors, and information gaps from the self-collected health data, (2) pinpoint relevant data and potential causes of noticeable medical events, and (3) recommend actions to follow to improve the reliability of the data issues and medical issues to be discussed with clinicians. The module uses a reasoning engine following a hypothesize-and-test strategy built on a knowledge base and using contextual information. The knowledge base contains hypotheses, rules, and plans we defined with the input of medical experts. We identified a large set of contextual information: emotional state (eg, preferences, mood) of patients and medical workers, their relationship, their metadata (eg, age, medical specialty), the time and location of usage of the system, patient-collected data (eg, blood glucose, basal-bolus insulin), patients’ goals and medical standards (eg, insulin sensitivity factor, in range values). Demonstrating the usage of the system revealed that (1) participants perceived the system as useful and relevant for consultation, and (2) the system uses less than 30 milliseconds to treat new cases.

**Conclusions:**

Using a knowledge-based system to identify anomalies concerning the reliability of patients’ self-collected health data to provide information on potential information gaps and to propose relevant medical subjects to discuss or actions to follow could ease the introduction of self-collected health data into consultation. Combining this reasoning engine and the system of the FullFlow project could improve the diagnostic process in health care.

## Introduction

### Background

Providing the right explanations regarding the situation of a patient at the right time is a key for improving the diagnostic process in health care [1]. Data collected by the patients, using various applications, can be a precious source of information for characterizing and explaining the situation of a patient suffering from chronic illnesses, especially diabetes [2], for both patients as well as their clinicians. Studies have shown that patients are increasingly using applications for automatically collecting, storing, and analyzing their data [3]. However, clinicians cannot effectively use self-collected health data until it is integrated into their daily workflow and clinical systems, and often ignore the data if they do not know that it is “accurate, reliable and aligned with their agenda” [4].

The “Full Flow of Health Data Between Patients and Health Care Systems,” referenced as FullFlow in this article proposes to address these issues. This can be achieved by providing a platform for integrating the patient’s self-collected health data from diabetes self-management applications (eg, Diabetesdagboka [5], mySugr [6]) and wearables (eg, FreeStyle Libre [7]) into Norwegian Electronic Health Records (EHRs) and Norwegian Personal Health Records (PHRs) through Norwegian public services. FullFlow aims to (1) facilitate diagnostic processes conducted by specialists, general practitioners (GPs), and nurses, by presenting patients’ self-collected health data directly in their EHRs and PHRs, and (2) empower patients and help them understand their disease. We limited the focus of FullFlow to diabetes, even if it can provide a more general service.

FullFlow consists of 3 components. First, there is a data collection component, which aggregates self-collected health data from the patients’ tools, by either using application programming interfaces (ie, automatic collection from patients’ tools) or Web-based schemas (ie, manual collection done by the patients). Second, there is a data analysis module, which processes the data and provides statistical analyses and medical calculations (eg, deviations, insulin sensitivity factor). Third, there is a Bundles Builder, which organizes the data into Fast Health Care Interoperability Resources (FHIR). FullFlow uses FHIR for facilitating its integration with Norwegian public services starting to implement this standard, especially Helsenorge.no [8], which contains a collection of health records generated by health care institutions (PDF only in May 2018) and accessible by both patients and clinicians in Norway. In addition to the FHIR-based data, the Bundles Builder provides reports to help medical workers consulting the data and to facilitate the integration of self-collected health data for the EHRs, which are not yet ready to handle FHIR resources but started to implement it [9]. These reports are dashboards, similar to the dashboard proposed by Dagliati et al [10] or to Carelink by Medtronic [11] but differs regarding several points: (1) FullFlow proposes the usage of self-collected health data as source of the dashboard, (2) FullFlow is aiming to integrate self-collected data into clinical systems directly without the use of external services, and (3) FullFlow is not limiting the data source to specific companies, sensors or applications. These reports are in PDF or Hypertext Markup Language and are directly sent to Norwegian EHRs and PHRs.

**Figure 1:**
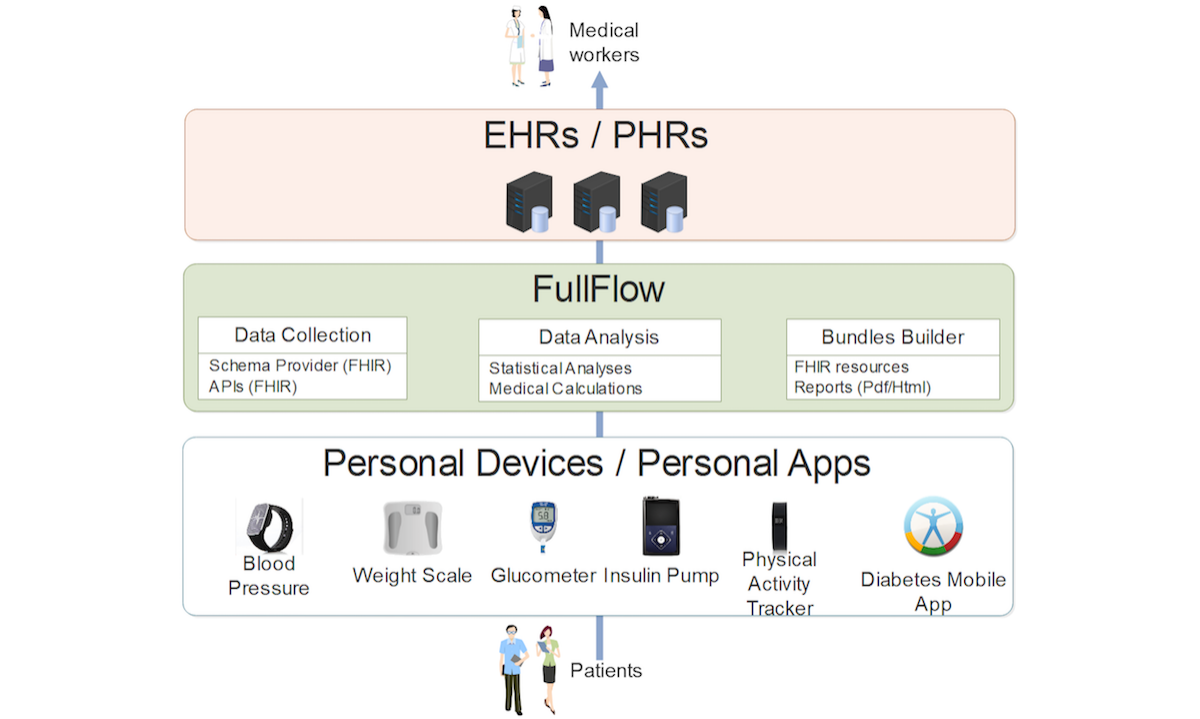
Simplified data flow of the FullFlow project. API: application programming interface; EHR: electronic health record; FHIR: Fast Health care Interoperability Resources; PHR: personal health record.

Figure 1 illustrates this composition and the data flow, from the patients to the medical workers.

**Figure 2:**
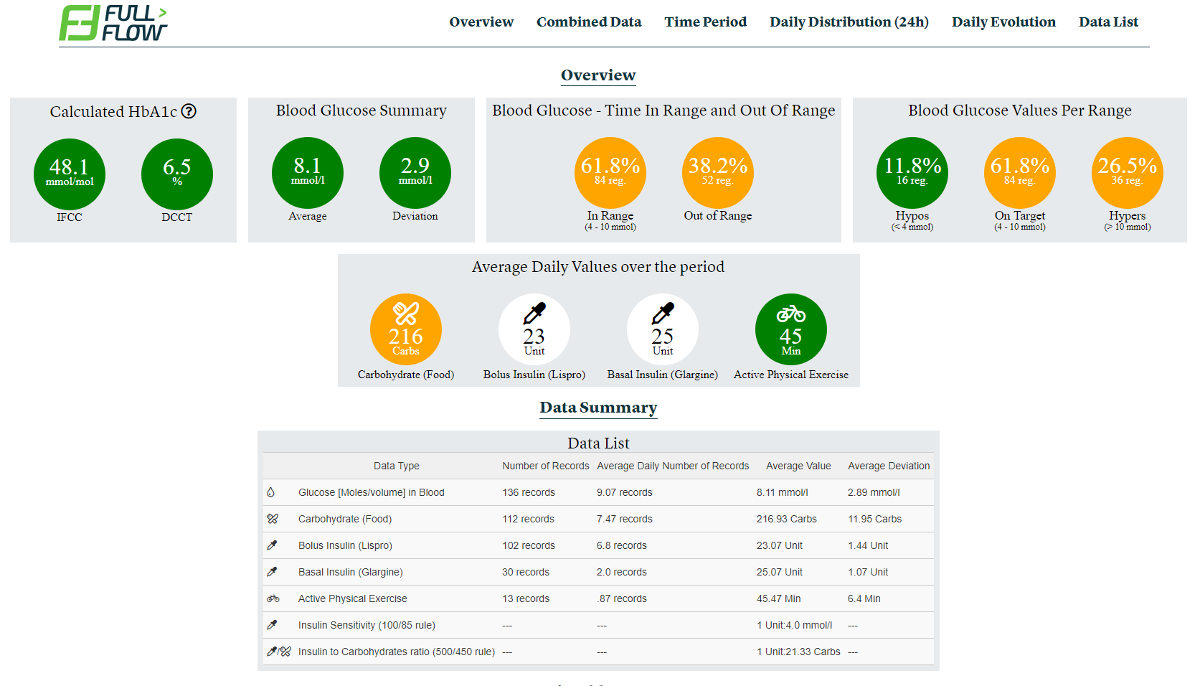
Example of a FullFlow Report.

The reports (see Figure 2) contain distinct areas, each focusing on a specific need:

1. Overview Area-provides a summary of the data period.
2. Period-displays patient-collected data as linear graphs.
3. Daily Evolution and Daily Distribution-contain graphs with all types of data available summarized per day and hour.
4. Data List-provides a list of all data collected for the period in text format.
5. Combined Data-displays all data in a unique graph.

These areas permit clinicians to obtain an overview of a patient’s self-reported health condition, as well as identify problematic events or trends, and to recommend actions for managing them. However, testing the dashboard of the FullFlow revealed unaddressed challenges.

First, the presence of information gaps in the self-collected health data. Information gaps are missing problematic events (eg, unreported hypoglycemic event) and lack of information for pointing out their causes (eg, undocumented extreme physical activity before a hypoglycemic event). Multiple factors lead to these information gaps (1) sensors and wearables used by the patients are not well calibrated, imprecise or even defective [12,13], (2) sensors and wearables are incorrectly operated by the patients [14], (3) patients make errors when registering data manually, and forget to register data or do not register at all [15], and (4) patients deliberately lie and edit the data to hide their poor performance to avoid unfavorable judgment by medical workers [16] and to avoid potential penalties. For example, in Norway, patients with more than 2 severe hypoglycemic events risk losing their driving license [17]. The information gaps limit the possibility for clinicians to interpret the data correctly and constitute the main barrier to the acceptance of the FullFlow, as the clinicians are considering the self-collected health data as less reliable compared to laboratory results for example.

Second, our workshops with clinicians showed that even when information gaps are not present, clinicians are unable to extract and analyze the data in an acceptable amount of time, especially during a consultation, even with the help of graphs. According to them, self-collected health data is too time consuming because of the amount of self-collected health data (ie, the number of registrations performed by the patients), of the noise in self-collected health data (ie, irrelevant data regarding the self-reported health condition of a patient), and clinicians need to link and compare different types of health data to extract information. This constitutes the second main barrier to the acceptance of the FullFlow.

**Figure 3:**
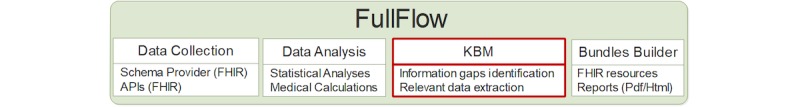
FullFlow components with the knowledge-based module (KBM; red). API: application programming interface; FHIR: Fast Health care Interoperability Resources.

In this paper, we address these challenges: information gaps, time-consuming processing of data and extraction of the relevance of the data by presenting the design, and implementation of a context-aware knowledge-based module (KBM). The KBM improves the FullFlow system by (1) providing information on the reliability of self-collected health data and the potential presence of information gaps, and (2) presenting relevant information about the self-reported health of a patient and the origins of problematic events.

The KBM is a complimentary module to dashboard systems such as FullFlow and could permit clinicians to focus on specific and relevant information during consultation instead of spending time consulting the self-collected health data and trying to extract information on their own. Figure 3 presents the FullFlow components with the KBM. The result section shows the impacts of the KBM on the Bundles Builder.

The knowledge base contains rules formulated by medical experts and relies on a reasoning engine (ie, component deducing information), based on contextual information, to identify and interpret relevant data. Dey and Abowd [18] define context as “any information that can be used to characterize the situation of an entity”. An entity is a person, place, or object that is considered relevant to the interaction between a user and an application, including the user and applications themselves. In our setting, medical evidence, such as patients’ self-collected health data, laboratory results and metadata, such as the identities of the patients and medical workers, and the rules of the knowledge base themselves compose the context. The reasoning engine combines these data using a hypothesize and test strategy for identifying data reliability problems as well as information gaps and highlighting relevant data related to problematic events.

This paper also presents the methodologies we followed from the creation to the assessment of this module, including its integration in the main system, and its future use.

## Methods

**Figure 4:**
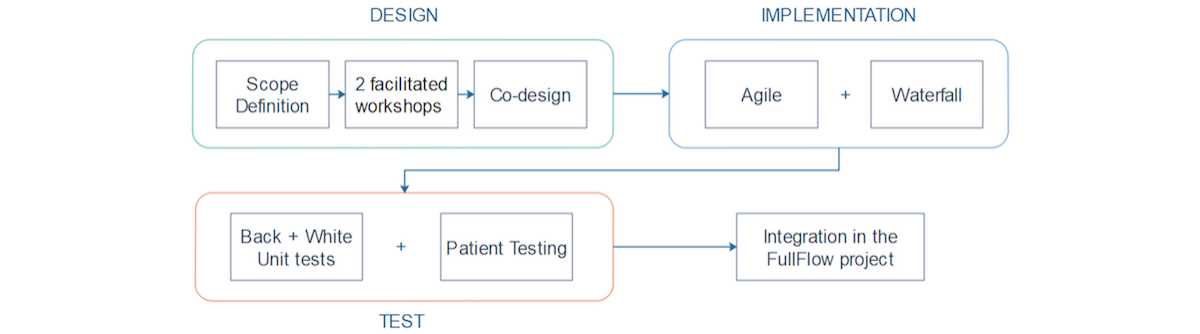
Methodologies used in three different phases: Designing, Implementing and Testing of the KBM.

This section presents an overview of the different phases and methodologies used for the design, the implementation and the testing of the KBM, as shown in Figure 4.

### Design of the Module

First, a brainstorming approach to define the scope of the module for identifying functionalities and potential problems appearing at a later stage was used by the main (AG) and the second author (PO). The data flow, technology stack (ie, a combination of programming languages, tools, and functionalities) and data model (ie, the standardization of data and relations between types of data) were also discussed.

Then, 2 facilitated workshops were organized for designing the KBM, involving the main author (AG), one patient with type 1 diabetes (in house researcher), and one clinician (AH). The workshops were used for different purposes (see Textbox 1). However, a wider range of people were invited to participate in a co-design workshop to contribute to the 3 points described above, as the 2 facilitated workshops sessions had limited participants. There were 5 patients with type 1 diabetes, 2 endocrinologists, and 2 nurses specializing in diabetes were involved in this co-design. The participants were not known to the authors and were recruited through the authors’ partner institution, the University Hospital of Northern Norway and on social media. Acknowledgment from Regional Ethical Committee was applied and an exemption was received September 2017. The co-design was organized around 3 sessions: (1) patients only, (2) clinicians only, and (3) all participants together. Sessions 1 and 2 were held simultaneously at a different location and before the session 3. This approach permitted to build the patients’ confidence and to ensure their thinking points were addressed during the common session. The patients’ pressure and bias were lowered by the facilitators (ie, the authors) giving everyone a chance to speak and by using different methodologies, such as (1) the expense account where each participant has to use a token before speaking and cannot speak once their token pile is empty, (2) the Writing Round Robin where all participants answer a question on paper simultaneously and then present the answers in turns, and (3) the 5 whys where a participant is asked “why” 5 times to find the root of a problem. The methodologies were defined beforehand by the authors through brainstorming sessions. Time was also reserved for participants to ask their questions throughout the sessions.

The co-design was audio recorded, and the audio registrations were transcribed by the authors for further classification and analysis. All medical related decisions from these events were assessed by the third author, who is a medical doctor.

### Implementation of the Module

An agile development process (ie, iterative development) was used for the software implementation when evolution, changes, and adaptability were the key points (eg, user interactions, reasoning model). Continuous input and involvement of patients and health workers were included in this process. A more classic waterfall approach (ie, sequential development) was used when stability and performance were the focus, such as the implementation of the core of the module (ie, the “engine” which does not interact directly with the users).

**The different purposes of the workshops.**

1. For identifying contextual information. The context was first identified following the approach proposed by Dey and Abowd [18] with the support of brainstorming: organizing context around location, identity, time and activity and using a tiered system for further categorization per type of context, and point of view of the KBM.
2. For creating a model of context, representing the interactions between all entities involved with the KBM (eg, patients, medical workers, EHRs) and the context interacted between them. This was inspired by the model of context in computer science proposed by Bradley and Dunlop [19] and was created to provide a complete overview of the usage of the context.
3. For defining a knowledge base and a reasoning model. They were used as requirements for the implementation of the module and to describe the functionalities of the KBM and its operation.

### Testing of the Module

Testing was performed in different ways: a white box (ie, testing of internal structures of code) approach was used for testing the core without involving the context and the reasoning model, while a black box (ie, testing of functionality) approach was followed for testing whether the system behaved according to what was defined by the previous creation process. Both approaches were made using unit tests. Load tests were used for determining if the performance of the modules could affect FullFlow in the event of its integration.

## Results

### System Architecture

This section presents a complete overview of the architecture of the KBM.

#### Contextual Information

The first step in the architectural design process (ie, the sequence of steps to create the KBM) was to identify the contextual information necessary for the KBM to achieve the goals for which it was designed. We adopted the context definition suggested in Dey and Abowd [18]. Their 4 main categories of context were location, identity, time, and activity. However, since the types of contextual information in health care domain is much richer than the context presented by Dey and Abowd, we introduced several types of context particularly instead of “activity” category of context.

**Figure 5:**
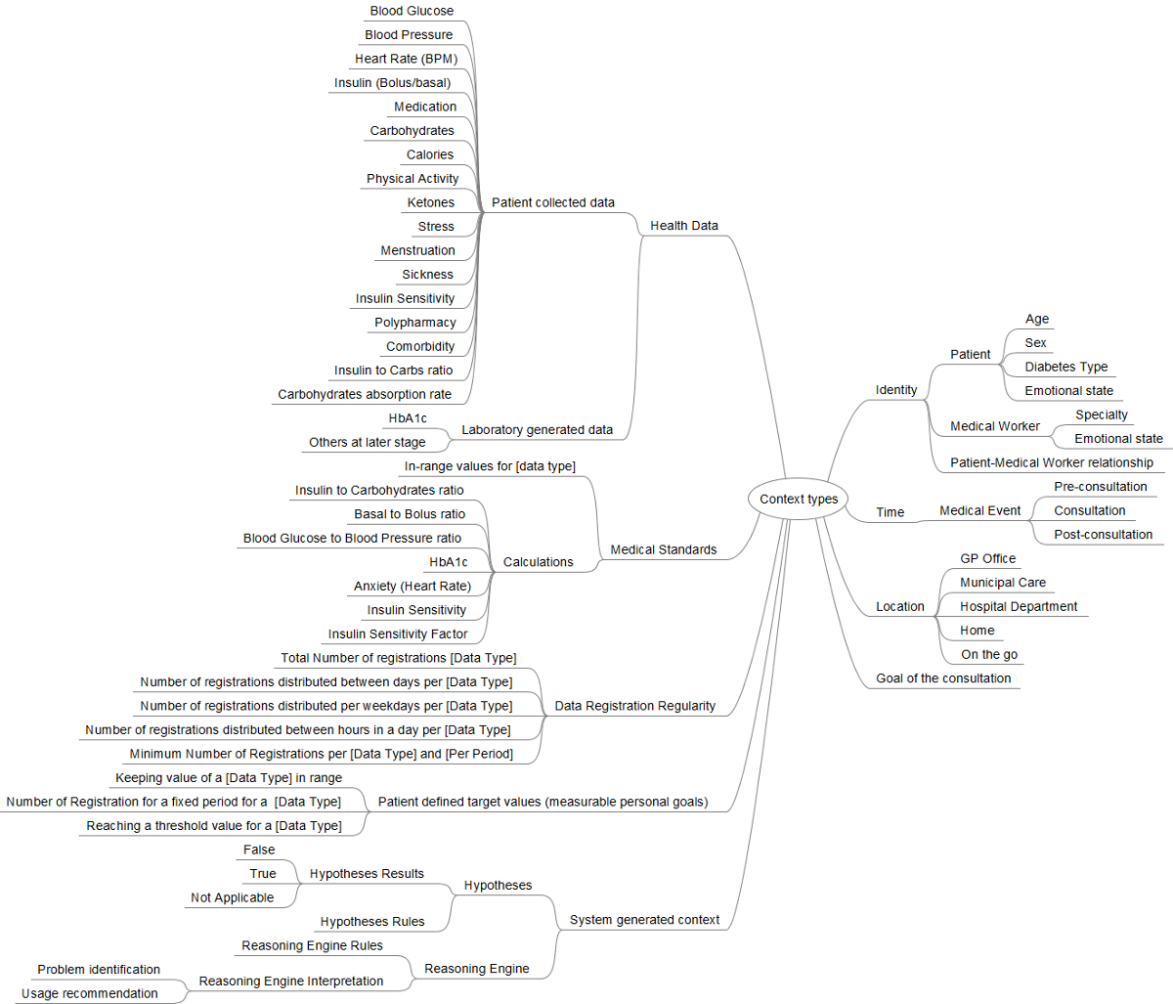
Categorization of Contextual Information Types.

In total, we identified 9 types of context, as shown in Figure 5. The first type is *health data*, containing *patient-collected data* and *laboratory generated data. Patient-collected data* represents data a patient can bring to the consultation using their sensors, mobile applications or diaries**.** The data usually collected by patients with diabetes are mostly blood glucose, basal-bolus insulin, carbohydrates, physical activity, and to a less degree also calories, blood pressure, heart rate, medication, ketones, stress, menstruation, sickness, insulin sensitivity, polypharmacy, comorbidity, insulin-to-carbohydrate ratio (I:C), and carbohydrate absorption rate. Units of measurements can further characterize each type of the collected data. For example, physical activity could be expressed as the number of steps, a period or intensity (eg, light, moderate, extreme), while insulin intakes could be expressed in international units (UI) or mg.

*Laboratory generated data* represents data originated from laboratory tests (eg, blood analysis). Today, FullFlow only has automatic access to the glycated hemoglobin (HbA_1c_) data from several EHRs and cannot obtain other types of data such as leukocytes, which are associated with diabetes complications [20], or creatinine which is useful for tracking the progression of diabetic kidney disease [21]. Therefore, they are not included in Figure 5.

*Medical standards* are the third type of context, which covers reference values for a specific data type. For example, the recommended range for blood ketones is less than 0.6 mmol/L or the formulae used for calculating medical values (eg, 1500/1800 rule for approximating the insulin sensitivity factor [22,23]).

*Data registration regularity* refers to the registration frequency for each type of data for different periods. The rationale behind this context type is to provide information on the regularity of measurements or samplings made by patients for each type of data they collect. The data registration contains the total number of registrations per self-collected data type for the whole period, as well as the distribution of the number of registrations per day, per weekday, and per hour, as well as a minimum number of registrations per data type and per period.

*Measurable personal goals* are the next type of context. Patients define them according to their preferred lifestyle or based on the feedback from their clinicians. There are several types of goals: (1) keeping the values of a specific data type within a specific target range (eg, keeping blood glucose between 4-9 mmol/L), (2) reaching a specific number of measurements for a fixed period (eg, checking blood glucose values 6 times a day with a glucose meter), and (3) reaching a threshold value for a specific data type (eg, weighing 65 kilograms or under).

*Goal of the consultation* refers to the reason for an appointment between a patient and the clinician. Clinicians can define the goal when planning a follow-up with patients, but patients can also define it if they need help regarding their health situation. The goal of the consultation may or may not be part of the patients’ diabetes situation.

*System generated context* refers to the context produced by the KBM itself during its execution. It includes hypotheses generated by the system that needs to be validated or refuted. The context *hypothesis result* further characterizes a hypothesis, with 3 possible states: (1) TRUE if the hypothesis is validated, (2) FALSE if the hypothesis is rejected, and (3) NOT APPLICABLE (NA) if the required context is missing (eg, the invalidation of a hypothesis stating that “the patient has eaten too much carbohydrates a day” cannot be done if the patient did not register any carbohydrate intake).

We identified 3 main entries under the *identity* type of context, which defines who uses the KBM in an actual situation. It encompasses patients, medical workers, and their relationship. Further context characterizes patients: age, sex, diabetes type. and emotional state (eg, personality, life goals, intentions, and preferences). Further context also characterizes clinicians: their specialty (eg, GP, nurse, endocrinologist) and their emotional state.

The *time* type of context *defines* when a patient and a medical worker use the KBM. In our situation, the usage of the module corresponds to the usage of the FullFlow system: mainly during consultations. However, medical workers and patients could also use it before and after consultation. In the first case, to prepare for the consultation, and in the second case, to look up data they did not have time to view during the consultation.

Concerning the *location* type of context, the KBM can be used everywhere: at a clinician’s workplace (eg, GP’s office, municipal care office, hospital department), at home or on the go for both patients and doctors, if they are willing to do so.

Instantiation of all these types of contextual information with the current situation where the KBM operates creates the “current context”. The current context is dynamic and changes across patients and different situation of the same patient (eg,, a particular consultation at a certain date and time and with a particular clinician for a particular purpose). In the section “Knowledge base and reasoning engine,” we describe the role of current context in the reasoning process of the reasoning engine.

#### Model of Context

The context taxonomy (ie, a classification scheme) in Figure 5 is the outcome of the first step of the design process. This has strong implications of the knowledge to be represented in the knowledge base as well. Context identification and modeling were performed by the designer group that consists of computer scientists and medical experts. There were 2 types of context predefined and do not change across situations: “Medical Standards” and “Data Registration Regularity”.

**Figure 6:**
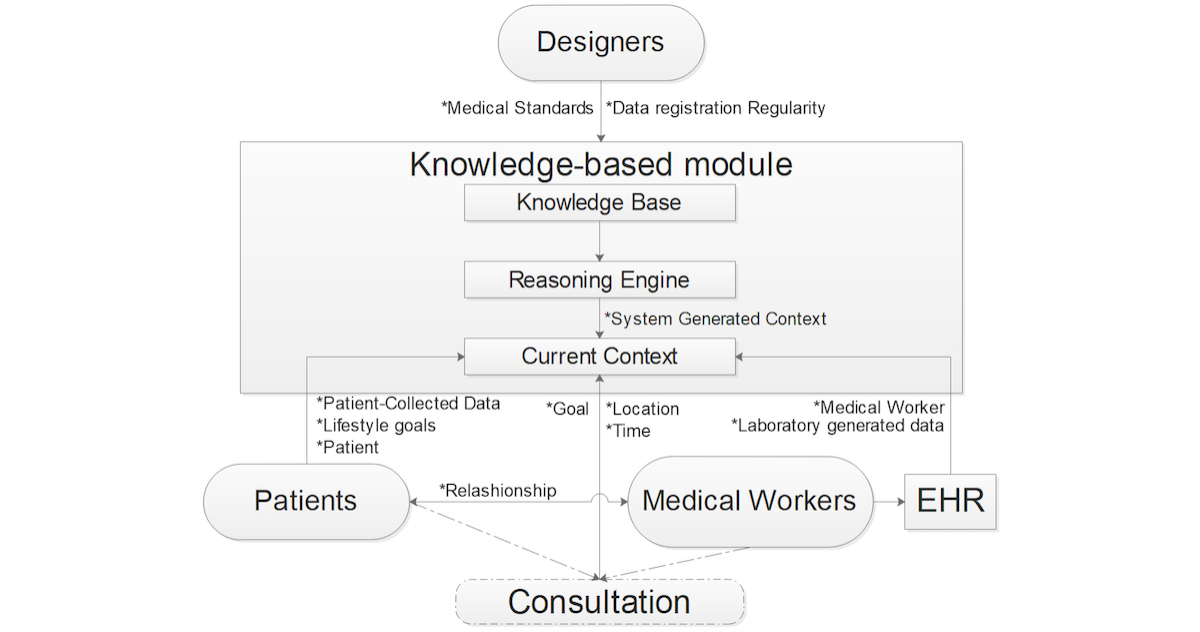
Model of Context. The labels next to the arrow represent the different types of context. EHR: electronic health record.

Once we identified the categories and the taxonomy of contextual information, we needed to define the interaction between entities (ie, the actors) and the specific part of the context shared during the interactions. To address this issue, we created a model of context inspired by the approach described by Bradley and Dunlop [19], as shown in Figure 6.

The *knowledge-based module* contains 3 components: the *knowledge base*, the *reasoning engine*, and the *current context*. There are 3 sources that create different parts of the current context—in addition to the designer defined ones. The first is patients. Patients interact with the module directly or through their PHRs (not displayed in the figure for simplicity) by sending their metadata (eg, age, sex, diabetes type) and self-collected health data. Second is medical workers and EHRs. Medical workers are not interacting directly with the KBM for sharing context, but through the EHRs they are using. EHRs provide the KBM with an authentication token for the medical workers in combination with the laboratory-generated data. Medical workers and patients interact with each other during a consultation, which could be face-to-face, remote, in real-time, or not. Third, is the reasoning engine. Outcomes of the reasoning engine of the KBM can dynamically change the current context. Here we refer to “system generated context” in Figure 5. For example, the original goal of the consultation could have been to discuss and manage nocturnal hypoglycemic events. However, the goal could shift toward discussing the insulin correction factor if the KBM finds that these events are due to wrong insulin dosage after meals, for example.

This context model allows us to have a clearer view of how the global flow of context data is in real-life situations.

#### Knowledge Base and Reasoning Engine

**Figure 7:**
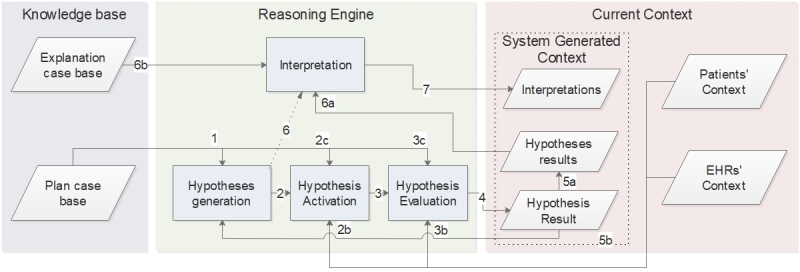
Our reasoning engine model.

We established the reasoning engine and the knowledge base by the identified types of contextual information and the model of context presented above. The reasoning engine provides problem-identifying functions needed for determining the degree of reliability of the patients’ self-collected health data and for identifying “noticeable events” and their potential causes. A noticeable event is a medical event discovered from the contextual information, where feedback from the medical worker could be useful for improving the patient’s situation. To do so, the reasoning engine relies on a knowledge base and a hypothesize-and-test reasoning strategy, as shown in Figure 7.

The rectangles in the figure represent the processes of the reasoning engine, while the parallelograms show the data the processes use or produce.

The *knowledge base* contains the domain knowledge of medical experts that the hypothesis-and-test strategy needs in this system. Currently, knowledge base remains static. Each time a patient shares their self-collected health data with a clinician, the same knowledge base creates the problem-identifying tasks, while the Current Context is dynamic. The *Explanation Case Base* and the *Plan Case Base* compose the KB.

**Figure 8:**
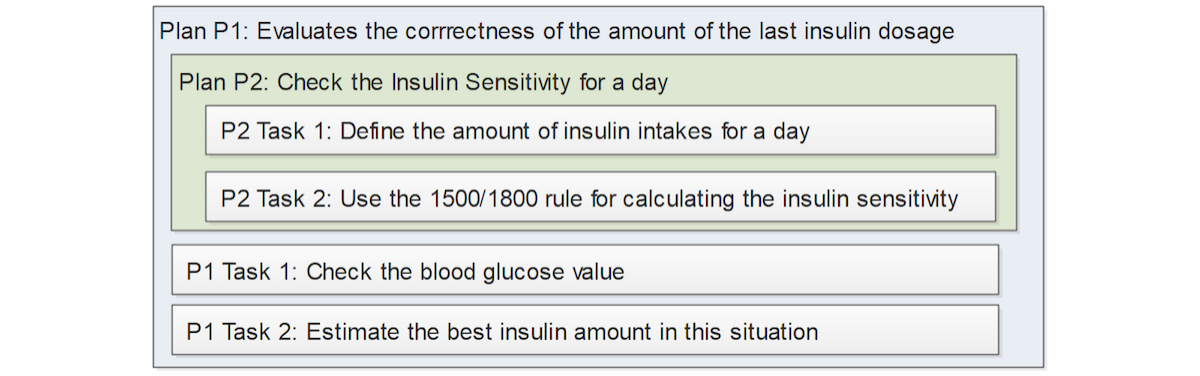
Example of hierarchy of plans (P) and tasks (T). P1 contains P2 and two tasks, P1T1 and P1T2.

We now describe the structure of the *Plan Case Base,* which comprises many plans*.* A plan consists of sequential problem-identifying tasks to perform and can refer to or include other plans. For example, plan P1 (ie, evaluates the correctness of the amount of the last insulin dosage) uses the tasks P1T1 (ie, check the blood glucose value), and P1T2 (ie, estimate the best insulin amount in this situation) in combination with the plan P2 (ie, check the insulin sensitivity for the day), which in turn includes the tasks P2T1 (ie, define the amount of insulin intakes for a day), and P2T2 (ie, use the 1500/1800 rule for calculating the insulin sensitivity). Figure 8 illustrates this example. This hierarchical structure, however, does not indicate in what sequence the tasks and plans are executed, but this is handled by *rules*.

There are 3 types of rules. The *Plan Rules* define the sequence of the plans and the tasks composing them (eg, perform the task “check if insulin registrations are present’ before the task ‘check the amount of insulin intake for a day”). The *Activation Rules* define which data are necessary for performing a task (eg, insulin and carbohydrates registrations are mandatory for the task “check if the patient forgot to take insulin before or after a meal”) and potential conditions for performing the task (eg, “a carbohydrate intake is considered a meal if done between 11:00 and 13:00”). The *Evaluation Rules* define the concrete actions to be taken in order to accomplish a task (eg, for the task “check if the patient forgot to take insulin before or after a meal,” the rules define 3 actions: (1) check the carbohydrates intakes, (2) check if the intakes correspond to a meal time, and (3) check if an insulin registration is present in a 30 minutes window before or after the carbohydrates intakes).

The *Explanation Case Base* defines the complementary or hierarchical relations between the problem-identifying tasks and the interpretation of identified problems based on the results of the problem-identifying tasks. For example, the problem-identifying tasks “check the amount of carbohydrate intake from the previous meal” and “calculate the carbohydrates on board” are complementary and compose the higher-level task “check the amount of carbohydrates”, which can characterize a hyperglycemic event.

The first process in the reasoning engine is *Hypotheses Generation*. In our model, a hypothesis represents the inferred candidate result of a task that the reasoning engine validates or invalidates. For example, the hypothesis “there is no insulin registration before or after a meal” may be a candidate answer to the task “check if the patient forgot to take insulin before or after a meal”. This process generates a current plan case composed of a sequence of tasks with associated hypotheses to test based on the plan and the Plan Rules of the Plan Case Base (Figure 7, no. 1) and on the System Generated Context (current context). The process uses the results of previously tested hypotheses to update the active case plan if necessary (Figure 7, no. 5b). For example, if the hypothesis “patient has hyperglycemia” is true, the process updates the plan and adds 18 hypotheses according to the rules, such as “the latest insulin intake was lower than the insulin needed defining by the sensitivity factor for reaching 5.5 mmol/L”. The outcome of the Hypotheses Generation is a sequence of hypotheses to validate (or refute), each for the accomplishment of a specific task constituting the plan.

The second process is *Hypothesis Activation*. The hypotheses generation process initiates this process for each hypothesis listed in the current plan case (Figure 7, no. 2). Hypothesis Activation requires the Activation Rules from the Plan Case Base (Figure 7, no. 2c) and the current context from Patients, EHRs or both (Figure 7, no. 2b). The *Hypothesis Activation* process ensures that the required context for evaluating a hypothesis is available. For example, the hypothesis “patient has hyperglycemia” requires Blood Glucose registrations from the Patient entity. If required context is not available for a hypothesis listed in the current plan case, the system flags the concerned hypothesis as NA. If the required context is available, the system activates the hypothesis. The activation of a hypothesis automatically initiates its evaluation (Figure 7, no. 3).

The *Hypothesis Evaluation* process validates or invalidates the claim proposed by the hypothesis. To do so, this process uses the Evaluation Rules of the Plan Case Base (Figure7, no. 3c) and the current context from Patients, EHRs or both (Figure7, no. 3b). The output of this process is a hypothesis result (Figure7, no. 4), which could be true, false, or NA. This output is then stored with the other hypotheses results (Figure7, no. 5a) and sent back to the Hypothesis Generation process (Figure7, no. 5b) for potential current plan case updates.

Once the Hypotheses Generation activated all hypotheses in its current plan case, it triggers the Interpretation process (Figure7, no. 6). This process uses the Relations between problem-identifying Tasks and their Explanations from the Explanation Case Base (Figure7, no. 6b) as well as the hypotheses results (Figure7, no. 6a) to create a textual interpretation of the results of the execution of the reasoning engine to allow users to consult it. The textual interpretation is the final context generated by the system (Figure7, no. 7). The system then displays the context to the users.

#### Hypotheses List

**Figure 9:**
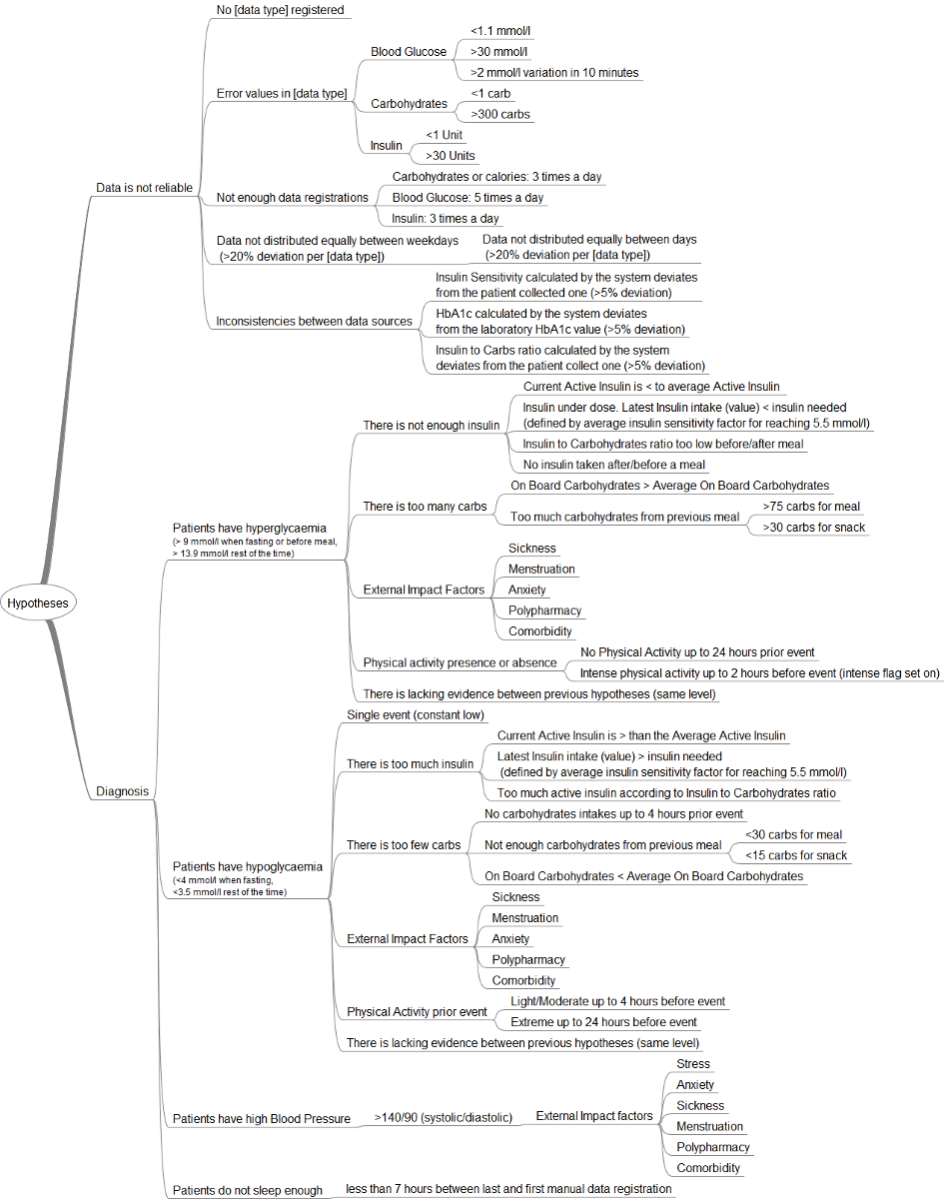
List of hypotheses organized per type used by the knowledge-based module (KBM).

Figure 9 describes all the hypotheses used by the KBM at this stage. We organized the hypotheses per type and per order of execution (ie, from top to bottom), according to the Explanation Case Base and of the Plan Case Base. The interpretation of the hypotheses defines them, instead of their internal identification code, for better clarity. For simplicity, we omitted the context requirements for their activation and generation in this paper. For example, the generation of the hypothesis “there is not enough insulin” requires that the hypothesis “patients have hyperglycemia” be true and its activation requires the registration of insulin self-collected health data.

##### Data Reliability

The first type of hypothesis relates to the *data reliability* of patients’ self-collected health data. The first hypothesis “data is not reliable” is automatically activated. The output of the evaluation process of this hypothesis is an impact factor of reliability, which defines how much the results of other hypotheses and the self-collected data can be trusted based on a scale of 0-50, from distrust to trust. The trust level is calculated by subtracting the sum of the value (or grade) of each sub-hypothesis evaluated to true by the system listed in the plan case of data reliability. For example, if the HbA_1c_ value calculated by the module (ie, based on blood glucose self-measurements) deviates by more than 5% (ie, based on the approximation of the translation of A1C to estimated average blood glucose by Nathan et al [24] and the inaccuracy of the blood-glucose monitoring systems for self-testing [25]) of the HbA_1c_ value determined by laboratory tests, the trust level decreases by 10 points. There are several types of sub-hypothesis. For example, “No [data type] registered” indicates that the most relevant data type is missing from the patient’s data: blood glucose, carbohydrates, insulin, and physical activity. Several sub-hypotheses compose this hypothesis: one per data type. For each hypothesis validated by the evaluation process (eg, “no blood glucose registered” is true), the interpretation process displays a message to users proposing that they register a new type of data with the support of examples. For example, if the patient is using insulin and the hypothesis “no carbohydrates registered” is true, the system displays “registering carbohydrate intakes will permit a better estimation of your insulin correction dosage as well as …and could help you reduce variation, ie, highs and lows of your blood glucose values”.

“Error values in [data type]” means that the registered values for a specific data type are probably incorrect. For example, a blood glucose value of 1.1 mmol/L is probably due to error either in the registration or measurement process. Importantly, blood glucose levels less than 1.1 mmol/l provoke neurological damages [26]. However, the KBM conveys a specific message to users regarding these events, in addition to grading the trust level of the data, for them to validate the origin of these values. Currently, the module focuses only on blood glucose, carbohydrates, and insulin values for this sub-hypothesis.

“Not enough data registrations” focuses on the minimal number of registrations per type of data and per day to calculate trends. For example, patients should check their blood glucose at least 5 times a day for this sub-hypothesis to be false. The National Institute for Health and Care Excellence (NICE) recommends self-testing blood glucose level at least four times a day [27], but we increased this number for better accuracy. The interpretation process also displays a motivational message to encourage patients to register data more often if some hypotheses are true.

“Data not distributed equally between days” concentrates on the regularity of the total number of registrations per day and per type of data for the whole data self-collection period. The participants suggested allowing 20% deviation in the number of registrations and days. The “Data not distributed equally between weekdays” follows the same principle but organizes the day per weekdays instead (eg, Monday, Tuesday.). These 2 hypotheses ensure that patients register data regularly and that the registrations are not impacted by their lifestyles (eg, working during the week and performing outdoor activities on the weekend).

“Inconsistencies between data source” is another hypothesis where the system checks the difference in the value of the same data type from different sources and allows 5% deviation between them. The module implements 3 sub-hypotheses. The first is checking the HbA_1c_ value calculated by the module itself against the same value determined by a laboratory test as explained previously. The second is checking the insulin sensitivity calculated by the module against the same value reported by the patient, and the last is checking the Insulin to Carbohydrates ratio (I:C) calculated by the module against the same value reported by the patient. The system alerts the user to this deviation with warning messages.

The evaluation of the previous hypotheses gives (1) an indication about the accuracy and the reliability of the self-collected health data for the clinicians, and (2) recommendations for improving the reliability of the data for the patients.

##### Medical Problem Identification

The second type of hypotheses relates to *medical problem identification*. The activation of these hypotheses depends on the value of the patients’ self-collected data and concerns hyperglycemia, hypoglycemia, high blood pressure events, and short sleeping patterns. The time of the highest blood glucose value in a continuous hyperglycemic event (6 hours maximum—suggested by the participants) and the time of the lowest blood glucose value in a continuous hypoglycemic event define a reference time where the possible causes could be easier to detect by the module.

###### Hyperglycaemia

In the case of hyperglycemia, Hypotheses Generation activates the hypothesis and set its result to true if it detects one or more blood glucose values greater than 9 mmol/L when fasting or before a meal (ie, if the information is available) or 13.9 mmol/L at other times of the day during a single continuous event. A single event is a continuous hyperglycemic event without blood glucose levels returning to the normal range. We chose a higher hyperglycemic level than the standard ones (eg, greater than 7mmol/L when fasting [27]) based the input of the co-design (see section “Relevance of the ” for more details).

Once a hyperglycemia event is detected, the system updates the plan case automatically and adds 5 sub-hypotheses. The first is “there is not enough insulin,” whose result is true by default and which the module tries to invalidate. To do so, the Hypotheses Generation activates 4 sub-hypotheses and all of them should be false or NA to invalidate the parent hypothesis. This includes the current active insulin is less than the average active insulin. Active insulin, or insulin on board (IOB), is the amount of insulin remaining active at a time in the body. The IOB calculation follows the Open Artificial Pancreas System (OpenAPS) approach [28]. A current IOB lower than the average IOB means that less insulin is present at this time, which could be a factor of the hyperglycemic event. Next, the dose of the last insulin shot was insufficient: the amount of the last insulin intake was insufficient for bringing the blood glucose value to 5.5 mmol/L. This is the mean value of the recommended range of blood glucose values defined by several guidelines [27,29]. The hypothesis evaluation process calculates how many units of insulin are necessary to bring the blood glucose value to this level based on the insulin sensitivity factor. If the insulin sensitivity factor is not provided by the patient, it is calculated by using the 1500/1800 rule [22,23]. Then, the I:C is too low if a meal was taken up to 4 hours (ie, one hour more than the time needed for the serum glucose level to return to near-fasting values in healthy patients [30]) prior to the hyperglycemic event. The hypothesis evaluation process checks if the amount of carbohydrates consumed are “covered” by a shot of insulin using the I:C provided by the patient. If unavailable, the hypothesis evaluation process uses the daily I:C calculated from the total carbohydrates and total rapid-acting insulin of the same day. If the patient did not register carbohydrate intakes, the system uses the 500/450 rule [23,31]. Finally, no insulin taken after or before a meal. The hypothesis evaluation process checks if there was an insulin injection before or after the meal (ie, 30 minutes window—decided by the participants) to compensate for the carbohydrate intake.

The second sub-hypothesis is “there are too much carbohydrates”. As with the last hypothesis, this hypothesis is true unless all sub-hypotheses are false or NA. First, there are greater carbohydrates on board (COB) than the average COB. COB is the amount of carbohydrates remaining unabsorbed at a time. The COB uses the carbohydrate absorption rate reported by the patient. Too much unabsorbed carbohydrates can lead to a hyperglycemic event. Second, for patients not following a low-carb diet, the last carbohydrate intake was greater than the recommendation: more than 75 carbs for a meal and more than 30 carbs for a snack [32]. The module uses standards mealtime by default (eg, lunchtime from 11:00 to 13:00) but patients can report them as well. As with the previous one, a too-high carbohydrate intake could lead to a hyperglycemic event if not planned.

The third sub-hypothesis is the presence of external factors, such as menstruation or polypharmacy. External factors can greatly affect the patient’s metabolism and render calculations difficult [33]. The system currently flags their presence in case other hypotheses fail to find potential causes of the hyperglycemic event.

The fourth sub-hypothesis is addressing the lack of physical activity to explain the hyperglycemic event and is set to true if patients did not engage in any physical activity up to 24 hours before the noticeable event happened (ie, blood glucose levels can be impacted by physical activity 24 hours after it ended [34]).

The last sub-hypothesis is “lack of evidence”. The hypothesis evaluation process checks if the module has identified possible causes of the hyperglycemic event based on the results of other hypotheses. If the system detects a possible cause, the hypothesis is false. However, it is true if all other hypotheses have false or NA results. Having a true result for this hypothesis means that a potential *information gap* is present at the time of this event, and the system informs the user and invites them to investigate the data around the time of this event.

###### Hypoglycemia

Regarding hypoglycemic events, the system follows the same approach. It activates the hypothesis and sets its result to true if it detects one or more blood glucose values lower than 4 mmol/L when fasting (ie, if the information is available) or 3.5 mmol/L at others time of the day during a single continuous event. We chose a lower hypoglycemic level than the standard ones (ie,less than 4 mmol/L when not fasting [27]) based the input of the co-design session. See section “Relevance of the ” for more details. Once a hypoglycemia event is detected, the system further activates 5 sub-hypotheses automatically. The first is “there is too much insulin,” whose result is true by default and which the module attempts to invalidate. To do so, it activates 3 more sub-hypotheses and all of them should be false or NA to invalidate the parent hypothesis. First, the current active insulin is greater than the average active insulin. Having a high amount of insulin could be the cause of a hypoglycemic event. Second, the last insulin injection was too high: the amount of the last insulin intake was greater than the requirements (based on the insulin sensitivity factor) for bringing the blood glucose value to 5.5 mmol/L (mean value of the recommended range of blood glucose values defined by several guidelines [27,29]). Third, the current active insulin is greater than required according to the I:C.

The second hypothesis is “there are too few carbohydrates”. This hypothesis is also true by default until invalidated by processing 2 sub-hypotheses. First, there was no carbohydrate intake up to 4 hours prior to the hypoglycemic event. This is one hour more than the time needed for the blood glucose level to return to near-fasting values in healthy patients [30]. Second, for patients not following a low-carb diet, the last carbohydrate intake was lower than the recommendation of less than 30 carbs for a meal or less than 15 carbs for a snack [32].

The third hypothesis concerns the presence of external factors and functions the same way as the hyperglycemic event.

The fourth hypothesis is about physical activity prior to the hypoglycemic event. The module automatically activates and process 2 sub-hypotheses. First, the patient engaged in light to moderate physical activity up to 4 hours prior to the hypoglycemic event. Light to moderate physical activity intensity can be expressed with an intensity tag (ie, text), in time (ie, less than 60 minutes—defined by the participants), in steps (ie, less than 3000 steps [35]) or in Metabolic Equivalent of Task unit (ie, less than 6 METs [36]). Second, the patient engaged in extreme physical activity up to 24 hours prior to the hypoglycemic event [34].

The last hypothesis activated addresses the lack of evidence for finding possible causes of a hypoglycemic event and functions in the same manner as its counterpart for a hyperglycemic event.

Regarding high blood pressure events, a hypothesis is activated and set to true automatically when high blood pressure is detected (ie, greater than 140/90 (systolic/diastolic) [37]). The sub-hypotheses then checks the presence or absence of external factors and function in the same manner as that for the hyperglycemia and hypoglycemic events.

The last hypothesis concerns the patient’s sleeping pattern. One hypothesis per night is activated and focuses on identifying the time elapsed between 2 registrations performed manually by the patient (ie, not done automatically by sensors). The hypothesis is set to true if there is less than the recommended 7-hour sleep period [38].

After a discussion, the designers decided to discard patient-defined target values as input for the hypotheses. For example, the detection of hyperglycemia and hypoglycemic events could rely on patient-defined goals focusing on maintaining a blood glucose range between 3.5-12 mmol/L instead of the value the module currently uses. However, these values override medical standards already defining these events and could potentially induce errors in medical workers. The designers discarded other contextual information such as ketones and heart rate for the first version of the module, as patients rarely measure ketones themselves compared to the other data, and heart rate not being available on the Diabetesdagboka or Mysgr applications.

The presence or absence of information gaps also evaluates the relevance of the data for the clinicians (ie, no information gap means reliable data). The identification of the potential causes of a problem could provide conversational topics for clinicians and a retrospective review of medical events for patients and clinicians.

### Testing

The goal of the testing phase was to ensure that the designed KBM module works, does not affect the performance of FullFlow and that participants of the workshops find the module useful during a consultation. All conditions were met, and the module was integrated into the FullFlow project.

Testing the relevance of the medical outcome of the module was out of scope at this stage and will be performed during the clinical study of the FullFlow project. The discussion section presents more details on the situation.

#### Technical Implementation and Performance Assessment

The implementation of the KBM relied on the reasoning engine model described in Figure 7 and follows the same processes and sequences. Black and white unit tests were performed against the KBM (see Methods section) to ensure that the KBM provides the services defined in the Knowledge base and reasoning engine section. The assessment of the performance of the KBM showed that the execution time is lower than 30 milliseconds with a typical load of data and, therefore, does not affect the performance of FullFlow. Details about the technical implementation, the tests performed and an excerpt of the results of one instance of the KBM are provided in Multimedia Appendix 1.

#### Relevance of the Module

**Figure 10:**
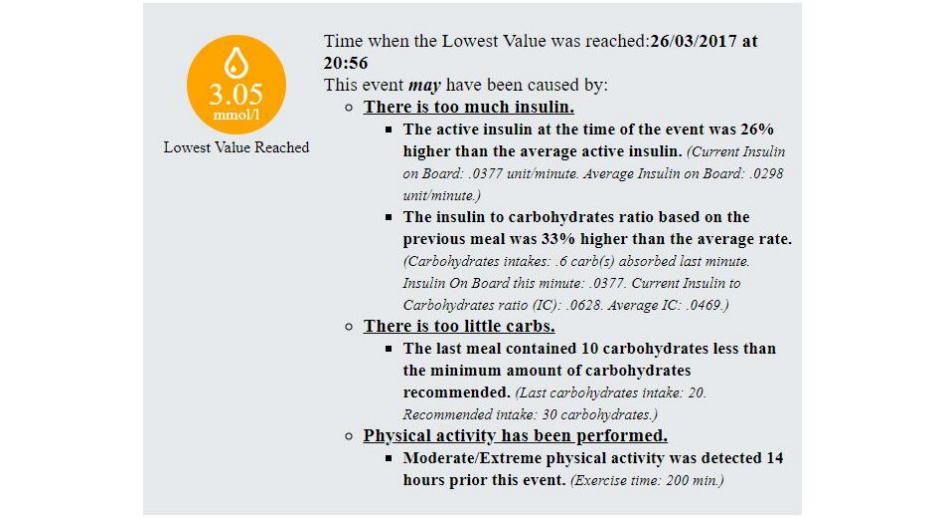
Example of potential causes expressed by the knowledge-based module of a single hypoglycaemic event.

**Figure 11:**
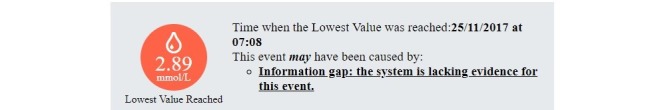
Example of information gap expressed by the KBM of a single hypoglycaemic event.

**Figure 12:**
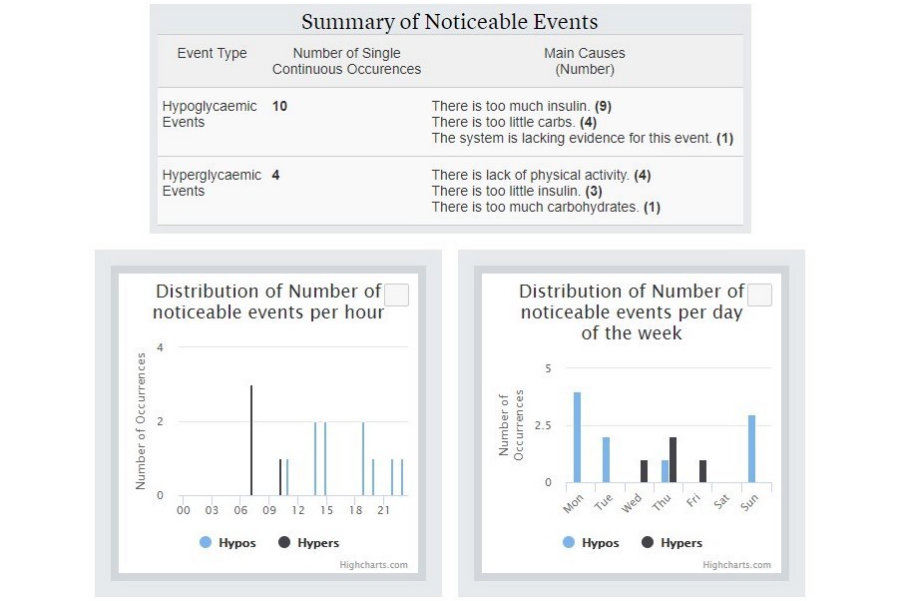
Summary of noticeable events detected by the knowledge-based module, their main potential main causes (top) and their distribution per hour and per weekdays (bottom).

**Figure 13:**
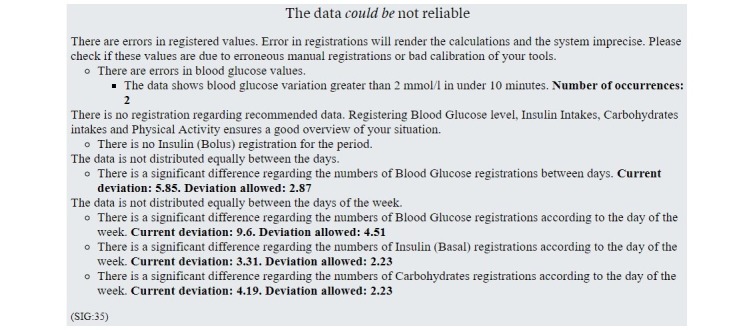
Summary of the data reliability issues found by the knowledge-based module.

We asked the participants of the clinician workshops and the co-design (ie, clinicians and patients) the same question: “do you think the module could be relevant during a consultation, especially for identifying potential problems?” and all of them answered yes. Then we showed the findings of the KBM within a FullFlow report to the participants. The findings are the results of a run of the KBM against self-collected health data provided by the in-house researcher. The results contained the noticeable events, their potential causes, and explanation, as well as their distributions through time, along with the reliability of the data (Figures 10, 11, 12, and 13 in the next section for more details).

There were 2 patients that preferred to have this module connected to their self-management solutions to (1) obtain suggestions on why serious medical events occur, and (2) to prepare for the consultation. The participants appreciated the concept of presenting the module between the overall view and the more detailed graphs in FullFlow because it permits faster identification of problems without having to examine the data. We discussed the KBM findings with the participants and how they felt about them. Based on these discussions the following actions were taken. First, we removed the data reliability grade from the visual display because it did not mean anything concrete to the participants. According to them, an alert stating the potential problems would be sufficient. Second, we changed the standards of hypoglycemia (ie, less than 5 mmol/L when fasting and less than 4 mmol/L at other times of the day) and hyperglycemia ie, greater than 7 mmol/L when fasting or before meals and greater than 9 mmol/L at other times) defined by the NICE [27] and the Norwegian Directorate of Health [29] to high hyperglycemia (ie, greater than 9 mmol/L when fasting or before meals and greater than 13.9 mmol/L) and low hypoglycemia (ie, less than 4 mmol/L when fasting and less than 3.5 mmol/L at other times) because the patients preferred to discuss the more serious events with their medical workers rather than all events outside the recommended range. Third, we updated the text displaying the feedback regarding medical events to be more nuanced (eg, “this event *may* have been due to…”) because the patients took for granted the findings of the module. However, in real life, we believe that medical workers also play a role here by limiting the impact on the patients.

Other than these points, the participants appreciated the module because it permitted them to obtain possible explanations for why events occurred and what they could improve.

Figure 10 shows an example of an Interpretation of the KBM regarding a hypoglycemic event. It this case, 4 potential causes were identified for explaining this event: (1) higher active insulin than average, (2) higher insulin to carbohydrates ratio, (3) presence of moderate or extreme physical activity before the event, and (4) a low-carbohydrates meal. The system provides justifications for all potential causes (ie, italic and smaller font text in the figure). Figure 11 shows an example of a representation of an information gap concerning a hypoglycemic event. Figure 12 shows a summary of noticeable events found by the KBM. It summarizes the number of hypoglycemic and hyperglycemic events (ie, 10 and 4 respectively) and the number of their potential main causes (eg, 9 hypoglycemic events may have been caused by having too much insulin). A single noticeable event can have multiple potential causes (eg, 14 potential causes are linked to 10 hypoglycemic events in the figure). The summary also contains a distribution per hour and per weekdays of the noticeable events. It can help clinicians identifying trend regarding daily or weekly routines followed by the patients.

Figure 13 shows a reliability grading of the self-collected health data. For example, the figure shows that there is a significant difference regarding the Blood Glucose registrations during the week, with a deviation of almost 6 registrations, while the rules allow a deviation of almost 3 registrations.

In 1 out of 14 (8%) noticeable events, the module lacked evidence to explain why a specific event occurred, which define an information gap. When discussing this with the patient concerned, he suggested that this could have been due to factors such as that he did not register, or estimated incorrectly carbohydrate intakes, for example.

The discussion showed that the module has a potential to improve the consultation between patients and clinicians and has, therefore, be integrated into the FullFlow.

## Discussion

### Demonstrated Potential

This paper demonstrated how a KBM using a hypothesize-and-test strategy fed with context may pinpoint the presence of information gaps in patient self-collected health data and identify relevant health information. It could address the barriers of acceptance regarding the introduction of patient self-collected health data into consultation: defining the reliability of the data and identify information gaps and reducing the necessary time for extracting the relevant information from the data. The recommendation of actions to follow to improve the self-collected data provided by the system could also motivate and empower patients by allowing them to be more aware of the possibilities offered by the technology. The suggestion of medical subjects related to the causes of medical events could also help steer the consultation and improve its efficiency.

### Likeliness for Use

We are aware that some patients could feel uncomfortable by a system judging them based on their disease management performance and their lifestyle. This could even be counterproductive for patients who are demotivated or make them less likely to adopt healthy self-managing routines, but using this system is intended to be voluntary and based on the patients deciding whether they want to gather and share data or not. We believe medical doctors could provide support to such patients and moderate the outcomes of modules like the one proposed during consultations. However, such patients are difficult to recruit for participation in studies for analyzing their needs, but we believe that by demonstrating the potential of such a system with examples like proposed in this paper, we will be able to recruit participants for the coming FullFlow project pilot. We also plan to organize workshops involving clinicians and psychologists focusing on motivation to address this issue.

### Chosen Approach

The hypothesize-and-test strategy is only 1 approach for inductive reasoning, which is the reasoning the module uses. For example, it was possible to use pattern recognition or machine learning to achieve the same goal. The key here concerns data acquisition and data sets. We do not possess high-quality patient self-collected health data at this time: insufficient patient diversity, insufficient patients, insufficient data distributed over long periods and the quality of the data itself could be doubtful because each patient is different and is focusing on different goals and using different applications. On top of that, the data could be erroneous as well. The strategy to acquire knowledge from experts can circumvent these issues, even if it is time-consuming and financially demanding.

### Limitations

First, the authors did not perform field-tests involving clinicians and patients in a real situation since the scope of this paper was to present and discuss the integration of the KBM into FullFlow.

Moreover, self-collected data represent only one source of data that could affect decision support and cannot replace other sources such as laboratory tests; above all, it cannot replace the relationship medical workers and patients have. Medical feedback concerning the module will be obtained during the clinical pilot of the FullFlow project, where patients and clinicians will be involved in a real consultation setting.

Third, we limited the focus of the KBM to patients with type 1 diabetes at this stage. However, the authors designed the reasoning engine model for supporting a multitude of medical conditions, especially patients with type 2 diabetes. An update of the knowledge base can adapt the KBM for patients with type 2 diabetes. The existing hypothesis “There is not enough insulin” can be activated only for patients with diabetes type 1 and for patients with diabetes type 2 on insulin therapy, while a new hypothesis “medication is not taken” can be created and activated for a patient with type 2 diabetes for example.

The system can exasperate medical workers if it does not support their needs or yields imprecise or erroneous information. However, as we defined the system with input from medical experts, we have reduced this risk.

The last point concerns that one patient only provided the self-collected health data. The target was to assess the relevance and usability of the module prior to possible integration into the FullFlow system, and subsequent trials will involve a larger number of patients and clinicians. The feedback provided by this patient and the participants in the workshops was used for justifying the KBM and prepare the FullFlow system for the main study.

### Dynamic Knowledge Base

At this stage, we decided to limit the scope of the KBM by keeping the knowledge base static for all situations, meaning that the system cannot create and interpret rules on its own. However, the reasoning engine model is dynamic and could support other diseases with an update of the knowledge base, as illustrated in the previous section. In addition, the inputs of the rules are dynamic, meaning that patients can provide their insulin to carbohydrates ratio or their mealtime to tailor the execution of the rules relying on these data. More dynamic inputs can be considered in the future such as measurable personal goals or recommendations from clinicians for example.

For the next iteration, we plan to use patients’ and clinicians’ context for generating the Plan Base Case and the Explanation Case Base to provide a more tailored experience for users, by using for example comorbidity as an input for generating the rules.

### Conclusion

To conclude, the hypothesize-and-test strategy is a viable approach for an inductive reasoning-based system when diverse and large and correct datasets are not available. The context-sensitive approach permits the integration of multiple factors for decision making and for simplifying the complexity and maintenance of this system.

By integrating this module to the FullFlow project, we hope to bring closer health institutions and self-managing patients, who do more on their own with seemingly less guidance from health institutions, by using the foundation for providing tailored health services during consultation: self-collected health data.

Our future clinical study will document user experience and medical outcomes through usage logs, interviews and medical and general surveys, and will help us adjust and improve this module further.

## Appendix

Multimedia Appendix 1 Implementation of the KBM.

## Abbreviations

API: application programming interface
COB: carbohydrates on board
EHR: electronic health record
FHIR: Fast Health care Interoperability Resources
GP: general practitioner
HbA _1c_: glycated haemoglobin
IOB: insulin on board
IU: international unit
KBM: knowledge-based module
MET: Metabolic Equivalent of Task
NA: not applicable
NICE: National Institute for Health and Care Excellence
PHR: personal health record
