# Supplementary material for: Design and Development of a Context-Aware Knowledge-Based Module for Identifying Relevant Information and Information Gaps in Patients With Type 1 Diabetes Self-Collected Health Data
Source: JMIR Diabetes. 2018 Jul 11;3(3):e10431. doi: 10.2196/10431 (PMC6238884; doi:10.2196/10431)
Supplement: Multimedia Appendix 1 [file diabetes_v3i3e10431_app1.pdf]

Appendix 1: Implementation of the KBM

This appendix presents the implementation and the performance testing of the KBM.

Implementation

The implementation relies on the same technologies used by the FullFlow server for easy integration: Java Enterprise edition (JEE) 8, Glassfish 5, Java Server Faces (JSF) 2.2 and the Java API for Extensible Markup Language (XML) Web Services (JAX-WS). However, we also wanted to allow execution of the KBM on Android OS, and integration with the Diabetesdagboka[1] application for future tests. We decided to use Plain Old Java Objects (POJOs) to represent most of the reasoning engine. This code is executable on both systems without requiring any supplementary work. However, the FullFlow server injects the current context and interpretation of the results to the KBM through contexts and dependency injection (CDI). For Android, it is necessary to use the DiabetesDagboka proprietary API for gathering the current context, and a string resource for collecting the interpretation. This section focuses on FullFlow implementation only, as shown in Figure 1.

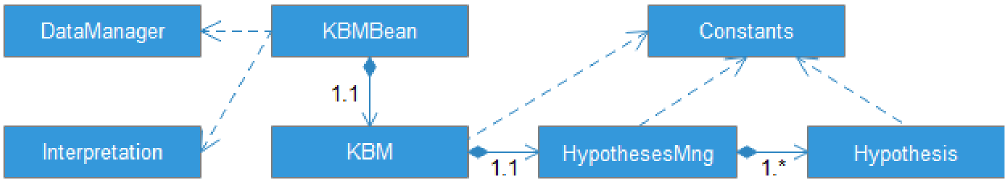

Figure 1: Simplified Unified Modeling Language (UML) diagram

The implementation is based on the reasoning engine model described in Figure 7 of the article “Context-aware knowledge-based module for identifying relevant information and information gaps in patients with type 1 diabetes’ self-collected health data” and follows the same processes and sequences, even if minor changes are observed. The Constants class (top right) contains the Plan Case Base of the system and the relation between the problem-identifying tasks, which is part of the Explanation Case Base. The Interpretation class (bottom left), which is a session bean, contains the interpretation of identified problems, which is another part of the Explanation Case Base. Splitting the Explanation Case Base permits multi-language management easily (in our case, English and Norwegian).

The DataManager class contains the current context collected by FullFlow. The HypothesesMng (Manager) represents the Hypotheses Generation process and keeps references to all hypotheses and their results. The Hypothesis class contains the Hypothesis Activation and Evaluation processes as well as the Hypothesis Result.

The KBM class represents the entire reasoning engine, initializing the Knowledge Base and the Current Context, and keeps references to all processes and data related to them. In addition, it contains the Interpretation process. We decided to include this process into this class for

simpler code maintenance and calls between classes, even if the model of reasoning engine separates them into two entities. The KBMBean exists as a bridge between the POJOs and the JEE environment.

The POJOs class share their execution status through interfaces. For example, a hypothesis calls a method, OnHypothesisResult(), which is implemented by the HypothesesMng to update the plan case. The HypothesesMng also calls a method, OnEvaluationPerformed(), for informing the KBM to initiate the interpretation process.

The reception of a request from a XML Web Services Interface or a patient visit on a JSF webpage initiates the flow of this system. The KBMBean is created and is injected with the DataManager and Interpretation beans, and creates a single KBM object by giving it all of the context available as parameters, which then perform the tasks described in the results section of the article.

Figure 2 shows an excerpt of the KBM execution results.

```
###### KBM START ######
KB_LOAD ToE:1084ns
-KBE_LOAD ToE:341ns
-KBP_LOAD ToE:292ns
-EXT_C_LOAD ToE:451ns
**********************************
******D: Patient: M [redacted] T1 831R
**********************************
**** HM HYP RUN ****
HYP_ID_DR:TRUE PT:42 ToE:292ns
-HYP_ID_DR_EV:TRUE ToE:292ns
--HYP_ID_DR_EV_BG:FALSE ToE:3507ns
---HYP_ID_DR_EV_BG_LESS:FALSE ToE:28055ns
---HYP_ID_DR_EV_BG_MORE:FALSE ToE:7890ns
---HYP_ID_DR_EV_BG_VAR:FALSE ToE:467580ns
[...]
--HYP_ID_DR_EV_INS:TRUE ToE:585ns
---HYP_ID_DR_EV_INS_LESS:TRUE ToE:26886ns
---HYP_ID_DR_EV_INS_MORE:FALSE ToE:6137ns
[...]
-HYP_ID_DR_NOT_ENOUGH:TRUE ToE:584ns
--HYP_ID_DR_NOT_ENOUGH_BG:FALSE ToE:64292ns
--HYP_ID_DR_NOT_ENOUGH_INS:FALSE ToE:15488ns
--HYP_ID_DR_NOT_ENOUGH_CARBS:TRUE ToE:14612ns
[...]
-HYP_ID_DR_DIST_DAILY:FALSE ToE:2046ns
--HYP_ID_DR_DIST_DAILY_15074-8:FALSE ToE:7154850ns
--HYP_ID_DR_DIST_DAILY_388454007:FALSE ToE:22795ns
--HYP_ID_DR_DIST_DAILY_411529005:FALSE ToE:9060ns
--HYP_ID_DR_DIST_DAILY_86047003:FALSE ToE:8475ns
[...]
-HYP_ID_DR_DIST_WEEKDAYS:FALSE ToE:1461ns
--HYP_ID_DR_DIST_WEEKDAYS_15074-8:FALSE ToE:14320ns
--HYP_ID_DR_DIST_WEEKDAYS_388454007:FALSE ToE:6430ns
--HYP_ID_DR_DIST_WEEKDAYS_411529005:FALSE ToE:4383ns
--HYP_ID_DR_DIST_WEEKDAYS_86047003:FALSE ToE:4675ns
[...]
-HYP_ID_DR_INC:NA ToE:293ns
--HYP_ID_DR_INC_HBA1C:NA ToE:6430ns
--HYP_ID_DR_INC_INS:NA ToE:4091ns
[...]
*** DIAG ***
HYP_ID_DIAG:TRUE ToE:22210ns
-HYP_ID_DIAG_HYPO:TRUE ToE:1168ns
************* Event From:16/12 2:28 to 16/12 2:28 BG:2.2222222222222223 ToE:584ns
--HYP_ID_DIAG_HYPO_SE:TRUE ToE:584ns
--CHANGE_OF_PLAN_INITIATED:LOAD_NEW_PLAN ToE:295ns
---HYP_ID_DIAG_HYPO_SE_TOO_MUCH_INS:TRUE ToE:585ns
----HYP_ID_DIAG_HYPO_SE_TOO_MUCH_INS_ACT:FALSE ToE:37114ns
----HYP_ID_DIAG_HYPO_SE_TOO_MUCH_INS_FACTOR:TRUE ToE:22502ns
----HYP_ID_DIAG_HYPO_SE_TOO_MUCH_INS_CARBS_RATIO:TRUE ToE:242264ns
---HYP_ID_DIAG_HYPO_SE_TOO_LITTLE_CARBS:TRUE ToE:292ns
----HYP_ID_DIAG_HYPO_SE_TOO_LITTLE_CARBS_NO:FALSE ToE:21626ns
----HYP_ID_DIAG_HYPO_SE_TOO_LITTLE_CARBS_INF:TRUE ToE:21918ns
---HYP_ID_DIAG_HYPO_SE_EXT:FALSE ToE:293ns
----HYP_ID_DIAG_HYPO_SE_EXT_STRESS:FALSE ToE:5845ns
----HYP_ID_DIAG_HYPO_SE_EXT_MENS:FALSE ToE:2338ns
----HYP_ID_DIAG_HYPO_SE_EXT_SICKNESS:FALSE ToE:2338ns
----HYP_ID_DIAG_HYPO_SE_EXT_PHARM:FALSE ToE:2046ns
----HYP_ID_DIAG_HYPO_SE_EXT_COMORBID:FALSE ToE:1753ns
---HYP_ID_DIAG_HYPO_SE_PA:FALSE ToE:292ns
----HYP_ID_DIAG_HYPO_SE_PA_LIGHT:FALSE ToE:7598ns
----HYP_ID_DIAG_HYPO_SE_PA_EXT:FALSE ToE:4384ns
---HYP_LACK_OF_EVIDENCE:FALSE ToE:5553ns
*************
[...]
-HYP_ID_HIGH_BP:FALSE ToE:8182ns
-HYP_ID_NOT_ENOUGHT_SLEEP:TRUE NB_N:18/20 ToE:4209095ns
**** HM HYP RUN END ToE:25464019ns ****
###### KBM END ToE:25465103ns ######
```

Figure 2: Excerpt of the results of one instance of the KBM. ToE = Time of execution

Performance testing

Before measuring the performance of the module, unit tests supporting the white and black box approaches (see Methods section of the article for more details) were performed against the KBM POJOs (Plain Old Java Objects, used in reasoning engine). The unit testing framework Junit

[2] was used for all tests. An example of a unit test for verifying that the KBM detects a variation greater than 2 mmol/L blood glucose in an interval of less than 10 minutes is shown in Figure 3.

```
@Test
public void verifyBloodGlucoseVarianceDetection(){
    Hypothesis h = new Hypothesis();
    Calendar c = Calendar.getInstance();
    long ref = c.getTimeInMillis(); //now
    c.add(Calendar.MINUTE,9);
    long low = c.getTimeInMillis(); //9 minutes in the future
    c.add(Calendar.MINUTE,2);
    long high = c.getTimeInMillis();//11 minutes in the future (9+2)
    assertEquals(Result.FALSE, h.bgVar(2.1, 4.1,ref,low), "<2mmol/l");
    assertEquals(Result.TRUE, h.bgVar(2.1, 4.2,ref,low), ">2mmol/l & <10min");
    assertEquals(Result.FALSE, h.bgVar(2.1, 5,ref,high), ">10min");
    assertEquals(Result.FALSE, h.bgVar(2.1, 4.1,ref,high), ">10min & <2mmol/l");
}
```

Figure 3: Example of a Unit Test

Then, to measure the potential impact on the performance of the FullFlow system, the time of execution for each method of the POJOs (KBM, HypothesesMng and Hypothesis classes; see Figure 1) were measured using nanoseconds. The Figure 2 shows the result of an excerpt run. However, the nanoseconds timing can be affected by the optimization of the Java Virtual Machine (JVM), the GlassFish server as well as the system host. To address this issue, these tests were performed on a fresh Debian 9 installation, with JVM 8 (Oracle) and Glassfish 5, with no customization (e.g. default heap space, default domain) and on a local computer (i7-7800X @3.50 GHz). The second problem of this test is that the current context will affect the performance. For example, more blood glucose registrations mean more time for calculating the deviation, and no insulin registrations mean no evaluation process for some hypotheses. To address this issue, we simulated a typical load for the module: 3 weeks of self-collected health data. According to the authors and participants of the workshops, 3 weeks could represent the average time between consultations when patients are actively addressing health issues. A super-user of the Diabetesdagboka (Diabetes Diary) app provided the self-collected health data. We define a super-user as a patient with diabetes who is extremely active in self-management and who register data regularly.

Figure 2 shows an excerpt of the KBM results and its time of execution. In total, less than 26 milliseconds (ms) were necessary for the KBM to perform its task as shown in line 70. In fact, the majority of the methods were executed in less than 1 ms. This is mainly due to the KBM focusing on applying rules on looked-up context already provided by FullFlow, with some exceptions. The next paragraph describes the most important parts of the excerpt.

Lines 2–5 show the creation of the KBM class, with the creation of the Explanation Case Base and the first version of the current plan case, based on the Plan Base Case, with the loading of the current context. This task took less than 1 ms (1084 nanoseconds).

Line 7 shows the case to be treated by the KBM. The patient is male, has type 1 diabetes and registered 831 records in his diary application over 3 weeks. This represents almost 40

registrations per day. For comparison, *reliable* patients included in our previous studies registered their health data between 10 and 20 times per day.

The remaining lines show the time of execution of each hypothesis according to the plan defined by the KBM class and executed by the HypothesesMng class. See Section Hypotheses List for an explanation on the calculation done by the hypotheses.

Lines 10–41 concerns all hypotheses under the category 'data is not reliable', and are part of the current plan defined by the KBM. The reliability grade obtained by this run is 42 out of 50, as shown in line 10, and means that the data is reliable. Two methods took more than 1 ms to be executed: HYP_ID_DR_EV_BG_VAR (line 15, 5 ms) and HYP_ID_DR_DIST_DAILY_15074-8 (line 27, 7 ms). The first corresponds to verification of blood glucose variation, which should be under 2 mmol/L in 10 minutes, and the second corresponds to the daily distribution of the number of blood glucose registrations that should be under 20% deviation (15074-8 is the Logical Observation Identifiers Names and Codes [LOINC] code of glucose [moles/volume] in blood). This section of this excerpt shows that the patient did not register enough carbohydrates regularly (line 24, <1 ms), and registered erroneous insulin values lower than 1 unit (line 18, <1 ms), resulting in a penalty of 8 points of the data reliability score.

Lines 42–68 show an example of health problem-identifying hypotheses. Lines 44–64 show a hypoglycaemic event. According to line 45, this event occurred on the 16th December 2017 at 02:28. The *from* and *to* fields of line 45 are the same because a single blood glucose registration was made during this hypoglycaemic event. This would not have been the case if the patient was using a continuous glucose monitor (CGM), for example. The blood glucose values were stored in the application in mg/dL and the system converted it to mmol/L, which explains the value displayed in the same line. Line 47 shows that HypothesesMng updated the current plan case when it detected a hypoglycaemic event. Therefore, it added hypotheses to the plan, which correspond to lines 48–64.

These hypotheses show that this event could have been caused by excess insulin (line 48, <1 ms) based on the insulin factor calculation (line 50, <1 ms) and the I:C (line 51, <1 ms). It also shows that the last carbohydrate intake was lower than the recommended value (line 54, <1 ms). If the hypothesis on line 64 is true, that means there is a potential information gap regarding this event, as the module did not find any causes that could have led to this event.

Line 67 shows that the patient had correct blood pressure measurements (<1 ms) and line 68 shows that the patient did not sleep correctly according to recommendations on 18 out of 20 nights (4 ms).

This load test proved that the potential negative impacts on the FullFlow performance were insignificant

1. Årsand E. Diabetesdagboka. 2018-03-07; Available from: URL:http://www.diabetesdagboka.no/en/. Accessed: 2018-03-07. (Archived by WebCite® at http://www.webcitation.org/6xjkhnEJJ).
2. JUnit. 2018-03-07; Available from: URL:https://junit.org/junit5/. Accessed: 2018-03-07. (Archived by WebCite® at http://www.webcitation.org/6xjmaWo5N).
